# Supplementary material for: Relationship between diabetes self-care practices and control of periodontal disease among type2 diabetes patients in Bangladesh
Source: PLoS One. 2021 Apr 6;16(4):e0249011. doi: 10.1371/journal.pone.0249011 (PMC8023471; doi:10.1371/journal.pone.0249011)
Supplement: S1 Table — (DOCX) [file pone.0249011.s001.docx]

**S1 Table.** Adjusted odds ratio (AOR) for associations between measures of oral hygiene practices

according to prevalence of Periodontal disease among type 2 diabetics individuals.

| **Oral hygiene practices** | n (%) | Periodontal disease (n-379) | |
| --- | --- | --- | --- |
|  |  | AOR (95% CI) | *P*-value |
| **Knowledge**  Periodontal disease can destroy teeth  No  Yes | 100 (26.4)  279 (73.6) | 1.00  1.40 (0.43-4.50) | 0.577 |
| Periodontal disease can affect overall health  No  Yes | 120 (31.7)  259 (68.3) | 1.00  0.81 (0.30-2.22) | 0.682 |
| Regular toothbrush can recede periodontal disease  No  Yes | 182 (48.0)  197 (52.0) | 1.00  0.35 (0.19-0.66) | 0.001 |
| Consumption of carbonated beverages can increase the risk  No  Yes | 187 (49.3)  192 (50.7) | 1.00  0.52 (0.28-0.98) | 0.044 |
| Consumption of fruits & vegetables can prevent periodontal disease  No  Yes | 187 (49.3)  192 (50.7) | 1.00  0.95 (0.50-1.81) | 0.870 |
| People with diabetes are more likely to have periodontal diseases  No  Yes | 153 (40.4)  226 (59.6) | 1.00  0.78 (0.40-1.52) | 0.466 |
| **Attitudes**  Important to take care of owns teeth  No  Yes | 42 (11.1)  337 (88.9) | 1.00  1.18 (0.22-6.39) | 0.850 |
| Needs to visit dentists regularly for periodontal disease  No  Yes | 52 (13.7)  327 (86.3) | 1.00  0.71 (0.10-5.09) | 0.737 |
| Regular and correct tooth brushing is necessary to prevent gum disease  No  Yes | 101 (26.6)  279 (73.4) | 1.00  0.38 (0.15-0.92) | 0.032 |
| Avoiding smoking is necessary to protect teeth from periodontal diseases  No  Yes | 96 (25.3)  283 (74.7) | 1.00  0.56 (0.17-1.85) | 0.343 |
| It is necessary for the diabetic health care provider to recommend the oral health examination for the diabetic patients  No  Yes | 80 (21.1)  299 (78.9) | 1.00  1.05 (0.31-3.56) | 0.940 |
| [Eating a healthy diet](https://www.deltadentalins.com/oral_health/nutrition.html) is necessary to prevent periodontal disease  No  Yes | 113 (29.8)  266 (70.2) | 1.00  1.02 (0.42-2.41) | 0.997 |
| **Behaviours**  Per day cleaning teeth frequency, times  <2  ≥2 | 160 (42.2)  219 (57.8) | 1.00  0.76 (0.43-1.33) | 0.337 |
| Brushing time, in minute  <2  ≥2 | 171 (45.1)  208 (54.9) | 1.00  0.44 (0.24-0.83) | 0.011 |
| Types of cleansing aid used  Others*^1^*  Toothbrush | 42 (11.1)  337 (88.9) | 1.00  0.54 (0.12-2.41) | 0.419 |

(Continued…….)

| **Oral hygiene practices** | n (%) | Periodontal disease  (n-379) | |
| --- | --- | --- | --- |
|  |  | AOR (95% CI) | P-value |
| Materials used to clean teeth*^2^*  Others*^3^*  Toothpaste/Tooth powder | 42 (11.1)  337 (88.9) | --- | -- |
| Changing toothbrush frequency  >6 months*^4^*  Anytime when it damaged/Within 3-6 months | 63 (16.6)  316 (83.4) | 1.00  1.40 (0.46-4.20) | 0.553 |
| Types of toothpaste used  Not fluoridated*^5^*  Fluoridated | 279 (73.6)  100 (26.4) | 1.00  0.18 (0.09-0.35) | <0.001 |
| Mouth rinsing eating afterwards  No  Yes | 86 (22.7)  293 (77.3) | 1.00  0.25 (0.11-0.60) | 0.002 |
| Tongue cleaning after meal or at the time of brushing  No  Yes | 184 (48.5)  195 (51.5) | 1.00  0.63 (0.37-1.09) | 0.099 |
| Per day frequency of eating candy/chocolate/sweets, times  ≥ 2  0-1 | 46 (12.1)  333 (87.9) | 1.00  0.48 (0.21-1.12) | 0.089 |

Note: *^1^* Others: fingers, branches of trees; *^2^* Materials used to clean teeth did not include in the multivariate regression model because of similarity of the respondents of types of cleansing aid used; *^3^* Others: coal or ash; *^4^* >6 months: (n=42) respondents not using toothbrush were included in this category; *^5^* Not fluoridated: (n=42) respondents not using toothbrush were included in this category; Models were adjusted by age, sex, education, types of family structure, currently married, area of residence, and centre.
